# Brief Report: A population-based study of the impact of the COVID-19 pandemic on benzodiazepine use among children and young adults

**DOI:** 10.1007/s00787-024-02531-6

**Published:** 2024-08-07

**Authors:** Tony Antoniou, Kathleen Pajer, William Gardner, Melanie Penner, Yona Lunsky, Daniel McCormack, Mina Tadrous, Muhammad Mamdani, Peter Gozdyra, David N. Juurlink, Tara Gomes

**Affiliations:** 1 Li Ka Shing Knowledge Institute, St. Michael’s Hospital, Toronto, ON Canada; 2 ICES, Toronto, ON Canada; 3 Department of Family and Community Medicine, University of Toronto, Toronto, ON Canada; 4 Department of Family and Community Medicine, St. Michael’s Hospital, Toronto, ON Canada; 5 Children’s Hospital of Eastern Ontario Research Institute, Ottawa, ON Canada; 6 Department of Psychiatry, University of Ottawa, Ottawa, ON Canada; 7 School of Epidemiology and Public Health, University of Ottawa, Ottawa, ON Canada; 8 ICES, Ottawa, ON Canada; 9 Autism Research Centre, Bloorview Research Institute, Holland Bloorview Kids Rehabilitation Hospital, Toronto, Canada; 10 Department of Pediatrics, University of Toronto, Toronto Ontario, Canada; 11 Azrieli Adult Neurodevelopmental Centre, Centre for Addiction and Mental Health, Toronto, ON Canada; 12 Department of Psychiatry, University of Toronto, Toronto, ON Canada; 13 Leslie Dan Faculty of Pharmacy, University of Toronto, Toronto, ON Canada; 14 Li Ka Shing Centre for Healthcare Analytics Research & Training, Unity Health Toronto, Toronto, ON Canada; 15 Temerty Faculty of Medicine, University of Toronto, Toronto, ON Canada; 16 Institute of Health Policy, Management, and Evaluation, University of Toronto, Toronto, ON Canada; 17 Department of Medicine, University of Toronto, Toronto, ON Canada

**Keywords:** COVID-19, Benzodiazepines, Child, Adolescent, Prescriptions / statistics & numerical data

## Abstract

The COVID-19 pandemic was associated with increases in the prevalence of depression and anxiety among children and young adults. We studied whether the pandemic was associated with changes in prescription benzodiazepine use. We conducted a population-based study of benzodiazepine dispensing to children and young adults ≤ 24 years old between January 1, 2013, and June 30, 2022. We used structural break analyses to identify the pandemic month(s) when changes in prescription benzodiazepine dispensing occurred, and interrupted time series models to quantify changes in dispensing following the structural break and compare observed and expected benzodiazepine use. A structural break occurs where there is a sudden change in the trend of a time series. We observed an immediate decline in benzodiazepine dispensing of 23.6 per 100,000 (95% confidence interval [CI]: -33.6 to -21.2) associated with a structural break in April 2020, followed by a monthly decrease in the trend of 0.3 per 100,000 (95% CI: -0.74 to 0.14). Lower than expected benzodiazepine dispensing rates were observed each month of the pandemic from April 2020 onward, with relative percent differences ranging from − 7.4% (95% CI: -10.1% to – 4.7%) to -20.9% (95% CI: -23.2% to -18.6%). Results were generally similar in analyses stratified by sex, age, neighbourhood income quintile, and urban versus rural residence. Further research is required to understand the clinical implications of these findings and whether these trends were sustained with further follow-up.

**Supplementary Information:**

The online version contains supplementary material available at 10.1007/s00787-024-02531-6.

## Introduction

The COVID-19 pandemic has been associated with increases in the prevalence of mood and anxiety disorders among children and young adults in several studies. [1] However, findings from studies examining the effects of the pandemic on the mental health of children and young adults vary, with some studies finding no changes or improvements in some symptoms. [2] Less well studied is how the pandemic influenced patterns of prescription benzodiazepine use in children and young adults. In one study from Scandinavia, use of benzodiazepines in young adults between the ages of 20 and 24 did not increase during the first year of the pandemic, with decreased use noted among youths in Sweden. [3] Additional studies are required to characterize the impact of COVID-19 on benzodiazepine use in children and young adults, for several reasons. First, there is a lack of research supporting the effectiveness and long-term safety of benzodiazepines in children and young adults. Moreover, benzodiazepines have been associated with harm in children and young adults. [4] In addition, pandemic-associated changes in benzodiazepine use among children and young adults may be more likely in jurisdictions with extended periods of school closures and/or where health service use for anxiety disorders was observed to increase during the pandemic. In Ontario, there were several cycles of school openings and closures during the pandemic (see supplemental Table 1 appendix for timeline) with students experiencing the most frequent fully remote school closures in Canada, totalling approximately 220 days. [5, 6] In addition, outpatient visits for anxiety disorders among children and young adults increased during the first year of the pandemic, beginning in July 2020. [7] However, whether this increase corresponded to a change in benzodiazepine use is unknown. Accordingly, we conducted a population-based study of benzodiazepine dispensing to children and young adults ≤ 24 years old in Ontario, Canada, between January 1, 2013, and June 30, 2022.

## Methods

We used Ontario’s administrative health databases, linked using unique encrypted identifiers and analyzed at ICES (formerly known as the Institute for Clinical Evaluative Sciences). The use of data in this project was authorized under Sect. 45 of Ontario’s Personal Health Information Protection Act, which does not require review by a Research Ethics Board. We identified benzodiazepine prescriptions using the Narcotics Monitoring System database, which contains comprehensive records of benzodiazepine prescriptions dispensed from all community pharmacies in Ontario, regardless of payer. We excluded prescriptions for clobazam, which is used primarily for seizure disorders.

Our outcome was the monthly rate of benzodiazepine use per 100,000 children and young adults, defined as the number of individuals dispensed a benzodiazepine each month divided by the population of children and young adults aged 0 to 24 for that period. We used seasonally adjusted structural break analyses to identify the pandemic month(s) when changes in benzodiazepine dispensing occurred and interrupted time series analyses to quantify the immediate step change and change in monthly benzodiazepine dispensing following the structural break. A structural break is defined as an abrupt change or shift in the trend of the time series. [8] We also calculated expected benzodiazepine dispensing rates for the period following the structural break using data between January 2013 and the month preceding the structural break and determined the relative percent changes between the observed and predicted dispensing rates. All models included dummy variables for month to account for seasonality and a variable denoting the implementation of a publicly-funded program known as Ontario Health Insurance Plan Plus (OHIP+) that covered the prescription costs for all individuals aged 24 and under beginning in January 2018. We stratified analyses by sex, age category (10 to 14 years, 15 to 19 years, 20 to 24 years), neighbourhood income quintile and urban versus rural residence. We estimated all models using Newey-West standard errors to account for autocorrelation up to 12 lags and heteroscedasticity. All analyses were completed using Stata version 17.0 (StataCorp LLC, College Station, TX, USA) and EViews 12.

## Results

During the study period, 256,270 individuals aged 24 or younger were dispensed a benzodiazepine. Most were female (*n* = 160,196; 62.5%), and the median age was 20 years (interquartile range: 17 to 22 years). The demographic characteristics of benzodiazepine-treated children and young adults did not change appreciably between the pre- and post-pandemic periods (supplemental Table 2).

Structural break analyses identified a change in trend in January 2018, the month of OHIP + implementation, and April 2020, the month following the declaration of a public health emergency and school closures. Following interrupted time series modelling, there was an immediate decline in benzodiazepine dispensing of 23.6 per 100,000 (95% confidence interval [CI]: -33.6 to -21.2) associated with the April 2020 structural break, followed by a monthly decrease in the trend of 0.3 per 100,000 (95% CI: -0.74 to 0.14) (supplemental Fig. 1, Table 1). Lower than expected benzodiazepine dispensing rates were observed each month of the pandemic from April 2020 onward, with relative percent differences ranging from − 7.4% (95% CI: -10.1% to – 4.7%) to -20.9% (95% CI: -23.2% to -18.6%) (Supplemental Table 3). Results were similar in stratified analyses, with some variability in the timing of the structural breaks (Table 1, Supplemental Tables 4 to 15). In addition, a second break following the onset of the pandemic was identified in age-stratified analyses among the 10 to 14 year old and 15 to 19 year old strata. However, observed benzodiazepine dispensing rates generally exceeded predicted rates for children aged 10 to 14 from June 2021 onward (Supplemental Table 12; Supplemental Fig. 2).

**Table 1:** Changes in benzodiazepine dispensing in children and young adults *≤* 24 years of age following COVID-19 pandemic

	COVID Structural Break	Rates of benzodiazepine dispensing			Interrupted time series analysis	
	Structural break(s)	Rate per 100,000 in month preceding break	Rate per 100,000 in month following break	Rate per 100,000, June 2022	Change in dispensing rate per 100,000, first month following break	Monthly change in dispensing rate trend per 100,000 post-break
Overall	April 2020	231.86	194.99	205.52	-23.6 (-36.6 to -10.7)	-0.30 (-0.74 to 0.14)
Sex						
Female	April 2020	286.08	233.06	258.84	-28.7 (-46.8 to -10.6)	-0.13 (-0.85 to 0.59)
Male	April 2020	180.36	158.86	154.97	-18.8 (-27.4 to -10.2)	-0.47 (-0.64 to -0.30)
Age						
10 to 14	April 2020 Feb 2021	65.08	47.48	71.18	-15.7 (-20.7 to -10.8) [1]	0.22 (-0.33 to 0.76) [2]
15 to 19	March 2020 Aug 2020	291.49	243.49	272.22	-67.2 (-105.2 to -29.2) [3]	-1.2 (-1.9 to -0.60) [4]
20 to 24	Jan 2021	597.16	565.12	556.63	-46.2 (-75.3 to -17.2)	-2.4 (-3.1 to -1.8)
Income Quintile						
1 (lowest)	June 2020	231.04	241.71	216.68	-5.8 (-17.8 to 6.2)	-1.1 (-1.4 to -0.82)
2	April 2020	241.50	206.71	214.70	-23.8 (-39.6 to -8.1)	-0.32 (-0.76 to 0.11)
3	April 2020	220.03	181.44	185.70	-24.1 (-37.5 to -10.7)	-0.38 (-0.90 to 0.14)
4	April 2020	218.6	179.6	201.46	-23.9 (-34.0 to -13.8)	0.01 (-0.30 to 0.32)
5	April 2020	237.2	183.0	213.86	-32.6 (-47.4 to -17.7)	0.00 (-0.61 to 0.62)
Rural vs. urban residence						
Rural	Sep 2020	219.9	224.9	217.13	1.6 (-10.8 to 13.9)	-1.3 (-1.8 to -0.82)
Urban	April 2020	232.4	194.9	204.91	-25.3 (-38.6 to -11.9)	-0.28 (-0.67 to 0.11)

^1^Immediate change associated with April 2020 break

^2^Monthly change in dispensing trend following February 2021 break

^3^Immediate change associated with March 2020 break

^4^Month change in dispensing trend following August 2020 break

## Discussion

Our study found an immediate decrease in benzodiazepine dispensing during the first two years of the COVID-19 pandemic. The decline in benzodiazepine use may reflect reduced mental health-related health service access among Ontario children and young adults during the early pandemic period, [7, 9] decreased use of these drugs for procedural anxiety with the transition to virtual health care, decreased prescribing for social and performance anxieties with the transition to virtual activities, no increase in the use of these drugs for anxiety for other indications, or increased prescribing of antidepressants for mood and anxiety disorders. [10] The additional breakpoints identified in the 10-to-14- and 15-to-19-year old strata may reflect the dynamic nature of the pandemic, with multiple rounds of school openings and closures and changes in patterns of health service use. Further research would be required to ascertain the specific events associated with breaks identified after the onset of the pandemic. Reasons for the slightly higher-than-expected use of benzodiazepines in children aged 10 to 14 also require further research, but may reflect pre-medication for ‘catch-up’ diagnostic and therapeutic procedures (e.g., bloodwork, endoscopy, etc.) resulting from delayed medical care and re-scheduled from the early pandemic period. Alternatively, this pattern may reflect an increased prevalence of anxiety in later months of the pandemic. [1, 3]

Strengths of our study include complete benzodiazepine dispensing data for all children and young adults in Ontario, regardless of insurance status, and a follow-up period of more than two years following the pandemic onset. Limitations include a lack of data regarding benzodiazepine indication. Further research is needed to understand whether these trends were sustained with further follow-up.

## Electronic supplementary material

Below is the link to the electronic supplementary material.

Supplementary Material 1

## Data Availability

The data set from this study is held securely in coded form at ICES. While data sharing agreements prohibit ICES from making the data set publicly available, access may be granted to those who meet pre-specified criteria for confidential access, available at https://www.ices.on.ca/DAS.
