# Supplementary material for: Brief Report: A population-based study of the impact of the COVID-19 pandemic on benzodiazepine use among children and young adults
Source: Eur Child Adolesc Psychiatry. 2024 Aug 7;34(2):791–4. doi: 10.1007/s00787-024-02531-6 (PMC11868134; doi:10.1007/s00787-024-02531-6)

**Supplemental Table 1: Timeline of Pandemic-Associated School Closures in Ontario, Canada**

| **Month** | **Timeline of School Closures and Reopenings** |
| --- | --- |
| 2020-03-14 | Schools closed |
| 2020-03-12 | First post-secondary school closure in province |
| 2020-09-08 | Public schools opened for 1st day of in-person learning; masks required for all staff and grade 4 to 12 students |
| 2021-01-04 | Public and private elementary and secondary school students moved to teacher-led remote learning (7 public health regions) |
| 2021-01-04 | Public and private secondary school students moved to teacher-led remote learning (27 public health regions) |
| 2021-01-12 | Mask use mandated indoors for grades 1 to 3, and outdoors for all students, with expanded testing and screening protocols |
| 2021-01-25 | Elementary/secondary schools resumed in-person learning with testing, asymptomatic screening and mask mandate for grades 1 to 3 (7 regions) |
| 2021-02-01 | Elementary and secondary schools resumed in-person learning (4 additional public health units) |
| 2021-02-08 | Elementary and secondary schools resumed in-person learning (13 additional public health units) |
| 2021-02-16 | Elementary and secondary schools resumed in-person learning in all 3 remaining public health units (Peel, Toronto and York Region) |
| 2021-04-19 | Public and private elementary and secondary schools moved to remote learning |
| 2021-09-07 | Schools open for the 2021–2022 school year; option of returning to in-person learning for full school day or synchronous remote learning |
| 2022-01-05 | All public and private school students resumed virtual learning, in-person return delayed |
| 2022-01-17 | Students returned to in-person learning |
| 2022-02-10 | Extra-curricular activities are allowed again in schools; students are required to wear a mask but may temporarily remove their mask for activities |

**Supplemental Table 2: Demographic and clinical characteristics of individuals aged 0 to 24 dispensed a benzodiazepine, January 2013 to June 2022**

| **Variable^a^** | **Entire Study Period (January 1, 2013 to June 30, 2022)** | **Pre-COVID (January 1, 2020 to March 31, 2020)** | **Post-COVID**  **(Apr 1, 2020-Jun 30, 2022)** | **Standardized Difference**  **Period 1-Period 2** |
| --- | --- | --- | --- | --- |
| Number of individuals | 256,270 | 218,299 | 66,193 |  |
| Age (median, IQR) | 20 (17-22) | 20 (17-22) | 20 (17-22) | 0.05 |
| 0-4 | 5,191 (2.0%) | 4,279 (2.0%) | 1,293 (2.0%) | 0.00 |
| 5-9 | 8,099 (3.2%) | 6,643 (3.0%) | 2,359 (3.6%) | 0.03 |
| 10-14 | 21,171 (8.3%) | 17,134 (7.8%) | 5,715 (8.6%) | 0.03 |
| 15-19 | 90,667 (35.4%) | 76,981 (35.3%) | 20,566 (31.1%) | 0.09 |
| 20-24 | 131,142 (51.2%) | 113,262 (51.9%) | 36,260 (54.8%) | 0.06 |
| Female, No. (%) | 160,196 (62.5%) | 136,091 (62.3%) | 42,598 (64.4%) | 0.04 |
| Income quintile |  |  |  |  |
| 1 (lowest) | 49,494 (19.3%) | 42,314 (19.4%) | 12,811 (19.4%) | 0.00 |
| 2 | 47,575 (18.6%) | 40,657 (18.6%) | 12,118 (18.3%) | 0.01 |
| 3 | 49,052 (19.1%) | 41,675 (19.1%) | 12,640 (19.1%) | 0.00 |
| 4 | 52,261 (20.4%) | 44,379 (20.3%) | 13,611 (20.6%) | 0.01 |
| 5 | 57,888 (22.6%) | 49,274 (22.6%) | 15,013 (22.7%) | 0.00 |
| Residence |  |  |  |  |
| Urban | 230,475 (89.9%) | 196,653 (90.1%) | 59,293 (89.6%) | 0.02 |
| Rural | 25,795 (10.1%) | 21,646 (9.9%) | 6,900 (10.4%) | 0.02 |
| Prescriber Type |  |  |  |  |
| General Practitioner | 156,321 (61.0%) | 135,218 (61.9%) | 37,289 (56.3%) | 0.11 |
| Pediatrician | 12,664 (4.9%) | 10,470 (4.8%) | 3,872 (5.8%) | 0.05 |
| Psychiatrist | 37,259 (14.5%) | 31,863 (14.6%) | 11,225 (17.0%) | 0.06 |
| Other | 50,026 (19.5%) | 40,748 (18.7%) | 13,807 (20.9%) | 0.06 |
| Average days’ supply of prescription (mean, SD) | 11.96 ± 11.71 | 12.30 ± 11.91 | 12.56 ± 12.23 | 0.02 |
| Days’ supply category |  |  |  |  |
| 1 to 7 | 119,726 (46.7%) | 99,221 (45.5%) | 30,308 (45.8%) | 0.01 |
| 8 to 14 | 58,141 (22.7%) | 49,671 (22.8%) | 14,523 (21.9%) | 0.02 |
| 15 to 29 | 38,732 (15.1%) | 33,985 (15.6%) | 9,691 (14.6%) | 0.03 |
| > 30 | 39,660 (15.5%) | 35,411 (16.2%) | 11,671 (17.6%) | 0.04 |
| Diagnosis in 30 days preceding dispensing date |  |  |  |  |
| Anxiety disorder | 120,703 (47.1%) | 102,397 (46.9%) | 29,026 (43.9%) | 0.06 |
| Mood disorder | 31,156 (12.2%) | 26,599 (12.2%) | 7,731 (11.7%) | 0.02 |
| Seizure disorder | 10,963 (4.3%) | 8,817 (4.0%) | 3,803 (5.7%) | 0.08 |

**Supplemental Table 3: Projected and Actual Benzodiazepine Dispensing in Children and Youth, April 2020 to June 2022**

| **Month** | **Projected Rate of benzodiazepine dispensing in absence of COVID pandemic (individuals per 100,000)** | **Actual Rate of benzodiazepine dispensing during COVID pandemic (individuals per 100,000)** | **Relative percent change, actual versus projected benzodiazepine dispensing (95% confidence interval** |
| --- | --- | --- | --- |
| April 2020 | 239.9 | 196.8 | -18.0% (-20.4% to -15.5%) |
| May 2020 | 236.5 | 195.0 | -17.5% (-20.0% to -15.1%) |
| June 2020 | 232.0 | 211.2 | -9.0% (-11.6% to -6.3%) |
| July 2020 | 229.6 | 212.7 | -7.4% (-10.1% to -4.7%) |
| August 2020 | 237.7 | 209.0 | -12.1% (-14.6% to -9.5%) |
| September 2020 | 233.5 | 215.7 | -7.6% (-10.3% to -4.9%) |
| October 2020 | 238.0 | 213.7 | -10.2% (-12.7% to -7.6%) |
| November 2020 | 234.2 | 210.2 | -10.2% (-12.8% to –7.6%) |
| December 2020 | 231.5 | 207.5 | -10.4% (-13.0% to -7.7%) |
| January 2021 | 243.0 | 211.8 | -12.8% (-15.3% to -10.3%) |
| February 2022 | 230.6 | 196.9 | -14.6% (-17.1% to -12.0%) |
| March 2021 | 245.6 | 221.0 | -10.0% (-12.6% to -7.5%) |
| April 2021 | 245.0 | 206.7 | -15.6% (-18.0% to -13.2%) |
| May 2021 | 241.5 | 210.2 | -13.0% (-15.5% to -10.5%) |
| June 2021 | 237.1 | 212.4 | -10.4% (-13.0% to -7.8%) |
| July 2021 | 234.7 | 203.0 | -13.5% (-16.0% to -11.0%) |
| August 2021 | 242.7 | 204.8 | -15.6% (-18.1% to -13.2%) |
| September 2021 | 238.5 | 212.6 | -10.9% (-13.4% to -8.3%) |
| October 2021 | 243.0 | 201.2 | -17.2% (-19.6% to -14.8%) |
| November 2021 | 239.2 | 208.0 | -13.0% (-15.5% to -10.5%) |
| December 2021 | 236.5 | 196.0 | -17.1% (-19.6% to -14.7%) |
| January 2022 | 248.0 | 196.9 | -20.6% (-22.9% to -18.2%) |
| February 2022 | 235.6 | 193.2 | -18.0% (-20.4% to -15.5%) |
| March 2022 | 250.7 | 214.4 | -14.5% (-16.9% to -12.0%) |
| April 2022 | 250.0 | 197.7 | -20.9% (-23.2% to -18.6%) |
| May 2022 | 246.6 | 196.8 | -20.2% (-22.5% to -17.8%) |
| June 2022 | 242.1 | 205.5 | -15.1% (-17.6% to -12.6%) |

**Supplemental Table 4: Projected and Actual Benzodiazepine Dispensing in Males 0 to 24 years of age, April 2020 to June 2022**

| **Month** | **Projected Rate of benzodiazepine dispensing in absence of COVID pandemic (individuals per 100,000)** | **Actual Rate of benzodiazepine dispensing during COVID pandemic (individuals per 100,000)** | **Relative percent change, actual versus projected benzodiazepine dispensing (95% confidence interval** |
| --- | --- | --- | --- |
| April 2020 | 186.0 | 156.9 | -15.7% (-19.5% to -11.7%) |
| May 2020 | 185.2 | 158.9 | -14.2% (-18.1% to -10.2%) |
| June 2020 | 180.7 | 167.5 | -7.3% (-11.5% to -3.0%) |
| July 2020 | 180.9 | 165.6 | -8.4% (-12.6% to -4.2%) |
| August 2020 | 184.0 | 158.3 | -14.0% (-17.9% to -9.9%) |
| September 2020 | 183.9 | 168.3 | -8.5% (-12.6% to -4.2%) |
| October 2020 | 184.4 | 162.9 | -11.6% (-15.7% to -7.5%) |
| November 2020 | 180.5 | 160.3 | -11.2% (-15.3% to -7.0%) |
| December 2020 | 181.4 | 159.7 | -11.9% (-16.0% to -7.8%) |
| January 2021 | 188.2 | 161.5 | -14.2% (-18.1% to -10.2%) |
| February 2022 | 177.7 | 148.9 | -16.2% (-20.1% to -12.1%) |
| March 2021 | 189.7 | 166.9 | -12.0% (-16.0% to -8.0%) |
| April 2021 | 188.0 | 156.8 | -16.6% (-20.4% to -12.7%) |
| May 2021 | 187.2 | 157.0 | -16.1% (-20.0% to -12.2%) |
| June 2021 | 182.7 | 159.2 | -12.9% (-16.9% to -8.8%) |
| July 2021 | 183.0 | 155.8 | -14.8% (-18.7% to -10.8%) |
| August 2021 | 186.0 | 156.8 | -15.7% (-19.6% to -11.8%) |
| September 2021 | 185.9 | 163.5 | -12.1% (-16.1% to -8.0%) |
| October 2021 | 186.5 | 154.9 | -16.9% (-20.7% to -13.0%) |
| November 2021 | 182.6 | 155.7 | -14.7% (-18.7% to -10.7%) |
| December 2021 | 183.4 | 150.7 | -17.8% (-21.7% to -13.9%) |
| January 2022 | 190.2 | 150.1 | -21.1% (-24.7% to -17.3%) |
| February 2022 | 179.7 | 146.5 | -18.5% (-22.3% to -14.5%) |
| March 2022 | 191.8 | 162.2 | -15.4% (-19.2% to -11.5%) |
| April 2022 | 190.1 | 148.0 | -22.1% (-25.8% to -18.4%) |
| May 2022 | 189.2 | 150.2 | -20.7% (-24.4% to -16.9%) |
| June 2022 | 184.7 | 155.0 | -16.1% (-20.0% to -12.1%) |

**Supplemental Table 5: Projected and Actual Benzodiazepine Dispensing in Females 0 to 24 years of age, April 2020 to June 2022**

| **Month** | **Projected Rate of benzodiazepine dispensing in absence of COVID pandemic (individuals per 100,000)** | **Actual Rate of benzodiazepine dispensing during COVID pandemic (individuals per 100,000)** | **Relative percent change, actual versus projected benzodiazepine dispensing (95% confidence interval** |
| --- | --- | --- | --- |
| April 2020 | 296.7 | 238.9 | -15.7% (-19.5% to -11.7%) |
| May 2020 | 290.5 | 233.1 | -14.2% (-18.1% to -10.2%) |
| June 2020 | 286.1 | 257.3 | -7.3% (-11.5% to -3.0%) |
| July 2020 | 280.9 | 262.2 | -8.4% (-12.6% to -4.2%) |
| August 2020 | 294.2 | 262.5 | -14.0% (-17.9% to -9.9%) |
| September 2020 | 285.7 | 265.6 | -8.5% (-12.6% to -4.2%) |
| October 2020 | 294.3 | 267.3 | -11.6% (-15.7% to -7.5%) |
| November 2020 | 290.6 | 262.8 | -11.2% (-15.3% to -7.0%) |
| December 2020 | 284.2 | 257.9 | -11.9% (-16.0% to -7.8%) |
| January 2021 | 300.7 | 264.9 | -14.2% (-18.1% to -10.2%) |
| February 2022 | 286.2 | 247.6 | -16.2% (-20.1% to -12.1%) |
| March 2021 | 304.5 | 278.1 | -12.0% (-16.0% to -8.0%) |
| April 2021 | 304.9 | 259.5 | -16.6% (-20.4% to -12.7%) |
| May 2021 | 298.7 | 266.4 | -16.1% (-20.0% to -12.2%) |
| June 2021 | 294.4 | 268.6 | -12.9% (-16.9% to -8.8%) |
| July 2021 | 289.1 | 252.7 | -14.8% (-18.7% to -10.8%) |
| August 2021 | 302.4 | 255.4 | -15.7% (-19.6% to -11.8%) |
| September 2021 | 293.9 | 264.4 | -12.1% (-16.1% to -8.0%) |
| October 2021 | 302.5 | 250.0 | -16.9% (-20.7% to -13.0%) |
| November 2021 | 298.8 | 263.2 | -14.7% (-18.7% to -10.7%) |
| December 2021 | 292.5 | 243.8 | -17.8% (-21.7% to -13.9%) |
| January 2022 | 308.9 | 246.3 | -21.1% (-24.7% to -17.3%) |
| February 2022 | 294.5 | 242.6 | -18.5% (-22.3% to -14.5%) |
| March 2022 | 312.8 | 269.4 | -15.4% (-19.2% to -11.5%) |
| April 2022 | 313.2 | 250.2 | -22.1% (-25.8% to -18.4%) |
| May 2022 | 307.0 | 245.9 | -20.7% (-24.4% to -16.9%) |
| June 2022 | 302.6 | 258.8 | -16.1% (-20.0% to -12.1%) |

**Supplemental Table 6: Projected and Actual Benzodiazepine Dispensing in Urban Residents 0 to 24 years of age, April 2020 to June 2022**

| **Month** | **Projected Rate of benzodiazepine dispensing in absence of COVID pandemic (individuals per 100,000)** | **Actual Rate of benzodiazepine dispensing during COVID pandemic (individuals per 100,000)** | **Relative percent change, actual versus projected benzodiazepine dispensing (95% confidence interval** |
| --- | --- | --- | --- |
| April 2020 | 240.6 | 196.4 | -18.4% (-20.9% to -15.8%) |
| May 2020 | 237.4 | 194.9 | -17.9% (-20.4% to -15.3%) |
| June 2020 | 233.0 | 211.0 | -9.5% (-12.2% to -6.7%) |
| July 2020 | 230.5 | 212.7 | -7.7% (-10.5% to -4.9%) |
| August 2020 | 238.4 | 208.5 | -12.5% (-15.2% to -9.8%) |
| September 2020 | 234.4 | 215.3 | -8.1% (-10.9% to -5.3%) |
| October 2020 | 238.5 | 213.3 | -10.6% (-13.3% to -7.8%) |
| November 2020 | 235.0 | 208.8 | -11.1% (-13.9% to -8.4%) |
| December 2020 | 231.8 | 205.9 | -11.2% (-13.9% to -8.4%) |
| January 2021 | 243.3 | 210.7 | -13.4% (-16.0% to -10.8%) |
| February 2022 | 231.1 | 196.2 | -15.1% (-17.7% to -12.4%) |
| March 2021 | 246.0 | 219.8 | -10.7% (-13.3% to -8.0%) |
| April 2021 | 245.5 | 205.0 | -16.5% (-19.0% to -13.9%) |
| May 2021 | 242.3 | 209.4 | -13.6% (-16.2% to -10.9%) |
| June 2021 | 237.9 | 211.4 | -11.1% (-13.8% to -8.4%) |
| July 2021 | 235.4 | 201.8 | -14.3% (-16.9% to -11.6%) |
| August 2021 | 243.3 | 203.8 | -16.2% (-18.8% to -13.7%) |
| September 2021 | 239.3 | 211.9 | -11.4% (-14.1% to -8.7%) |
| October 2021 | 243.3 | 200.7 | -17.5% (-20.0% to -14.9%) |
| November 2021 | 239.9 | 207.3 | -13.6% (-16.2% to -10.9%) |
| December 2021 | 236.7 | 194.6 | -17.8% (-20.3% to -15.2%) |
| January 2022 | 248.2 | 196.5 | -20.8% (-23.3% to -18.4%) |
| February 2022 | 236.0 | 193.9 | -17.8% (-20.4% to -15.2%) |
| March 2022 | 250.9 | 215.3 | -14.2% (-16.8% to -11.6%) |
| April 2022 | 250.4 | 198.2 | -20.8% (-23.3% to -18.4%) |
| May 2022 | 247.2 | 197.2 | -20.2% (-22.7% to -17.7%) |
| June 2022 | 242.8 | 204.9 | -15.6% (-18.2% to -13.0%) |

**Supplemental Table 7: Projected and Actual Benzodiazepine Dispensing in Rural Residents 0 to 24 years of age, September 2020 to June 2022**

| **Month** | **Projected Rate of benzodiazepine dispensing in absence of COVID pandemic (individuals per 100,000)** | **Actual Rate of benzodiazepine dispensing during COVID pandemic (individuals per 100,000)** | **Relative percent change, actual versus projected benzodiazepine dispensing (95% confidence interval** |
| --- | --- | --- | --- |
| September 2020 | 227.0 | 226.0 | -0.34% (-9.6% to 8.9%) |
| October 2020 | 235.5 | 224.9 | -4.5% (-13.3% to 4.4%) |
| November 2020 | 227.8 | 229.7 | 0.90% (-8.5% to 10.3%) |
| December 2020 | 230.6 | 228.5 | -0.89% (-10.1% to 8.3%) |
| January 2021 | 241.6 | 228.4 | -5.4% (-14.0% to 3.2%) |
| February 2022 | 227.2 | 209.3 | -7.8% (-16.5% to 0.91%) |
| March 2021 | 243.8 | 238.3 | -2.2% (-11.0% to 6.6%) |
| April 2021 | 238.4 | 228.6 | -4.0% (-12.8% to 4.7%) |
| May 2021 | 232.5 | 223.4 | -3.9% (-12.8% to 5.0%) |
| June 2021 | 230.3 | 227.6 | -1.1% (-10.2% to 8.0% |
| July 2021 | 229.1 | 220.4 | -3.8% (-12.7% to 5.2%) |
| August 2021 | 237.3 | 220.3 | -7.1% (-15.7% to 1.4%) |
| September 2021 | 232.9 | 224.7 | -3.5% (-12.4% to 5.4%) |
| October 2021 | 241.4 | 211.1 | -12.5% (-20.6% to -4.4%) |
| November 2021 | 233.7 | 220.9 | -5.4% (-14.2% to 3.3%) |
| December 2021 | 236.5 | 214.6 | -9.2% (-17.7% to -0.77%) |
| January 2022 | 247.5 | 206.8 | -16.4% (-24.2% to -8.7%) |
| February 2022 | 233.1 | 192.5 | -17.4% (-25.3% to -9.5%) |
| March 2022 | 249.7 | 212.2 | -15.0% (-22.8% to -7.1%) |
| April 2022 | 244.3 | 198.7 | -18.7% (-26.4% to -11.0%) |
| May 2022 | 238.4 | 198.1 | -16.9% (-24.8% to -8.9%) |
| June 2022 | 236.2 | 217.1 | -8.1% (-16.6% to 0.53%) |

**Supplemental Table 8: Projected and Actual Benzodiazepine Dispensing in Residents 0 to 24 years of age, Income Quintile 1, June 2020 to June 2022**

| **Month** | **Projected Rate of benzodiazepine dispensing in absence of COVID pandemic (individuals per 100,000)** | **Actual Rate of benzodiazepine dispensing during COVID pandemic (individuals per 100,000)** | **Relative percent change, actual versus projected benzodiazepine dispensing (95% confidence interval** |
| --- | --- | --- | --- |
| June 2020 | 246.5 | 244.4 | -0.83% (-7.1% to 5.4%) |
| July 2020 | 245.0 | 241.7 | -1.3% (-7.5% to 4.9%) |
| August 2020 | 248.0 | 234.3 | -5.5% (-11.4% to 0.63%) |
| September 2020 | 250.4 | 234.5 | -6.3% (-12.2% to -0.31%) |
| October 2020 | 251.9 | 230.3 | -8.6% (-14.3% to -2.7%) |
| November 2020 | 247.2 | 229.9 | -7.0% (-12.8% to -0.93%) |
| December 2020 | 239.1 | 226.0 | -5.5% (-11.5% to 0.75%) |
| January 2021 | 259.7 | 241.4 | -7.0% (-12.8% to -1.1%) |
| February 2022 | 243.9 | 230.0 | -5.7% (-11.7% to 0.49%) |
| March 2021 | 257.0 | 252.0 | -2.0% (-8.0% to 4.3%) |
| April 2021 | 255.8 | 235.1 | -8.1% (-13.8% to -2.2%) |
| May 2021 | 253.8 | 242.6 | -4.4% (-10.3% to 1.7%) |
| June 2021 | 249.2 | 2351 | -5.7% (-11.6% to 0.44%) |
| July 2021 | 247.7 | 224.5 | -9.3% (-15.1% to -3.4%) |
| August 2021 | 250.6 | 223.0 | -11.0% (-16.7% to -5.2%) |
| September 2021 | 253.2 | 231.0 | -8.8% (-14.5% to -2.8%) |
| October 2021 | 254.6 | 216.1 | -15.1% (-220.5% to -9.6%) |
| November 2021 | 249.9 | 232.4 | -7.0% (-12.8% to -0.98%) |
| December 2021 | 241.9 | 211.8 | -12.4% (-18.1% to -6.5%) |
| January 2022 | 262.4 | 217.1 | -17.2% (-22.5% to -11.8%) |
| February 2022 | 246.7 | 219.5 | -11.0% (-16.8% to -5.1%) |
| March 2022 | 259.7 | 235.8 | -9.2% (-14.9% to -2.4%) |
| April 2022 | 258.5 | 212.9 | -17.6% (-22.9% to -12.1%) |
| May 2022 | 256.5 | 213.0 | -16.9% (-22.3% to -11.4%0 |
| June 2022 | 252.0 | 216.7 | -14.0% (-19.6% to -8.2%) |

**Supplemental Table 9: Projected and Actual Benzodiazepine Dispensing in Residents 0 to 24 years of age, Income Quintile 2, April 2020 to June 2022**

| **Month** | **Projected Rate of benzodiazepine dispensing in absence of COVID pandemic (individuals per 100,000)** | **Actual Rate of benzodiazepine dispensing during COVID pandemic (individuals per 100,000)** | **Relative percent change, actual versus projected benzodiazepine dispensing (95% confidence interval** |
| --- | --- | --- | --- |
| April 2020 | 247.8 | 207.0 | -16.4% (-22.0% to -10.7%) |
| May 2020 | 243.4 | 206.7 | -15.0% (-20.7% to -9.2%) |
| June 2020 | 239.5 | 211.5 | -11.7% (-17.5% to -5.6%) |
| July 2020 | 237.5 | 221.4 | -6.7% (-12.9% to -0.40%) |
| August 2020 | 242.3 | 213.0 | -12.1% (-17.9% to -6.1%) |
| September 2020 | 242.2 | 224.0 | -7.5% (-13.5% to -1.3%) |
| October 2020 | 246.4 | 211.8 | -14.0% (-19.7% to -8.2%) |
| November 2020 | 239.3 | 217.1 | -9.3% (-15.3% to -3.1%) |
| December 2020 | 233.8 | 214.6 | -8.2% (-14.3% to -1.9%) |
| January 2021 | 251.5 | 218.4 | -13.1% (-18.8% to -7.3%) |
| February 2022 | 236.2 | 208.0 | -11.9% (-17.9% to -5.9%) |
| March 2021 | 255.4 | 235.1 | -7.9% (-13.8% to -1.9%) |
| April 2021 | 252.5 | 213.1 | -15.6% (-21.1% to -9.9%) |
| May 2021 | 248.0 | 211.4 | -14.7% (-20.4% to -8.9%) |
| June 2021 | 244.1 | 213.1 | -12.7% (-18.5% to -6.7%) |
| July 2021 | 242.1 | 208.6 | -13.8% (-19.6% to -7.9%) |
| August 2021 | 247.0 | 211.6 | -14.3% (-19.9% to -8.5%) |
| September 2021 | 246.9 | 224.9 | -8.9% (-14.8% to -2.8%) |
| October 2021 | 251.0 | 211.1 | -15.9% (-21.4% to -10.2%) |
| November 2021 | 244.0 | 210.8 | -13.6% (-19.3% to -7.7%) |
| December 2021 | 238.4 | 203.8 | -14.5% (-20.2% to -8.6%) |
| January 2022 | 256.1 | 203.3 | -20.6% (-25.9% to -15.2%) |
| February 2022 | 240.9 | 195.2 | -19.0% (-24.5% to -13.3%) |
| March 2022 | 247.8 | 229.4 | -7.4% (-13.4% to -1.2%) |
| April 2022 | 243.4 | 201.9 | -17.0% (-22.6% to -11.2%) |
| May 2022 | 239.5 | 198.7 | -17.0% (-22.7% to -11.1%) |
| June 2022 | 237.5 | 214.7 | -9.6% (-15.7% to -3.3%) |

**Supplemental Table 10: Projected and Actual Benzodiazepine Dispensing in Residents 0 to 24 years of age, Income Quintile 3, April 2020 to June 2022**

| **Month** | **Projected Rate of benzodiazepine dispensing in absence of COVID pandemic (individuals per 100,000)** | **Actual Rate of benzodiazepine dispensing during COVID pandemic (individuals per 100,000)** | **Relative percent change, actual versus projected benzodiazepine dispensing (95% confidence interval** |
| --- | --- | --- | --- |
| April 2020 | 227.0 | 179.1 | -21.1% (-26.4% to -15.6%) |
| May 2020 | 223.7 | 181.4 | -18.9% (-24.3% to -13.2%) |
| June 2020 | 218.3 | 196.4 | -10.0% (-16.0% to -3.9%) |
| July 2020 | 218.1 | 199.9 | -8.3% (-14.3% to -2.1%) |
| August 2020 | 225.8 | 197.7 | -12.4% (-18.1% to -6.5%) |
| September 2020 | 218.7 | 204.0 | -6.7% (-12.8% to -0.38%) |
| October 2020 | 223.8 | 202.5 | -9.5% (-15.4% to -3.4%) |
| November 2020 | 220.4 | 193.4 | -12.2% (-18.0% to -6.2%) |
| December 2020 | 216.6 | 189.6 | -12.4% (-18.3% to -6.4%) |
| January 2021 | 230.0 | 195.6 | -14.9% (-20.5% to -9.2%) |
| February 2022 | 220.1 | 182.8 | -17.0% (-22.6% to -11.2%) |
| March 2021 | 233.0 | 204.9 | -12.0% (-17.7% to -6.2%) |
| April 2021 | 232.0 | 191.7 | -17.3% (-22.8% to -11.8%) |
| May 2021 | 228.7 | 197.5 | -13.6% (-19.3% to -7.8%) |
| June 2021 | 223.4 | 199.2 | -10.8% (-16.7% to -4.8%) |
| July 2021 | 223.1 | 191.4 | -14.2% (-19.9% to -8.4%) |
| August 2021 | 230.8 | 192.0 | -16.8% (-22.3% to -11.2%) |
| September 2021 | 223.8 | 202.6 | -9.4% (-15.3% to -3.3%) |
| October 2021 | 228.8 | 186.6 | -18.4% (-23.8% to -12.9%) |
| November 2021 | 225.4 | 196.8 | -12.6% (-18.4% to -6.7%) |
| December 2021 | 221.7 | 182.1 | -17.9% (-23.4% to -12.2%) |
| January 2022 | 235.0 | 178.6 | -24.0% (-29.1% to -18.8%) |
| February 2022 | 225.2 | 174.0 | -22.7% (-28.0% to -17.3%) |
| March 2022 | 238.1 | 192.3 | -19.2% (-24.5% to -13.7%) |
| April 2022 | 237.1 | 181.8 | -23.3% (-28.4% to -18.0%) |
| May 2022 | 233.7 | 186.8 | -20.1% (-25.4% to -14.6%) |
| June 2022 | 228.4 | 185.7 | -18.7% (-24.1% to -13.0%) |

**Supplemental Table 11: Projected and Actual Benzodiazepine Dispensing in Residents 0 to 24 years of age, Income Quintile 4, April 2020 to June 2022**

| **Month** | **Projected Rate of benzodiazepine dispensing in absence of COVID pandemic (individuals per 100,000)** | **Actual Rate of benzodiazepine dispensing during COVID pandemic (individuals per 100,000)** | **Relative percent change, actual versus projected benzodiazepine dispensing (95% confidence interval** |
| --- | --- | --- | --- |
| April 2020 | 221.4 | 180.1 | -18.6% (-24.0% to -13.1%) |
| May 2020 | 219.5 | 179.6 | -18.1% (-23.6% to -12.6%) |
| June 2020 | 216.5 | 201.1 | -7.1% (-13.0% to -0.87%) |
| July 2020 | 211.3 | 195.8 | -7.3% (-13.4% to -1.1%) |
| August 2020 | 224.2 | 191.9 | -14.4% (-19.9% to -8.7%) |
| September 2020 | 219.5 | 205.2 | -6.5% (-12.5% to -0.36%) |
| October 2020 | 221.9 | 208.2 | -6.2% (-12.1% to -0.04%) |
| November 2020 | 219.1 | 198.8 | -9.2% (-15.1% to -3.2%) |
| December 2020 | 220.5 | 196.5 | -10.9% (-16.6% to -4.9%) |
| January 2021 | 226.1 | 194.7 | -13.9% (-19.4% to -8.2%) |
| February 2022 | 214.4 | 178.7 | -16.6% (-22.2% to -10.9%) |
| March 2021 | 228.5 | 199.7 | -12.6% (-18.1% to -6.8%) |
| April 2021 | 226.8 | 190.8 | -15.9% (-21.3% to -10.3%) |
| May 2021 | 224.9 | 193.3 | -14.1% (-19.6% to -8.4%) |
| June 2021 | 221.9 | 197.2 | -11.1% (-16.8% to -5.2%) |
| July 2021 | 216.7 | 192.0 | -11.4% (-17.2% to -5.5%) |
| August 2021 | 229.6 | 192.8 | -16.0% (-21.4% to -10.5%) |
| September 2021 | 224.9 | 198.6 | -11.7% (-17.3% to -5.9%) |
| October 2021 | 227.3 | 191.8 | -15.6% (-21.0% to -10.0%) |
| November 2021 | 224.5 | 196.2 | -12.6% (-18.2% to -6.8%) |
| December 2021 | 226.0 | 188.0 | -16.8% (-22.1% to -11.2%) |
| January 2022 | 231.5 | 191.5 | -17.2% (-22.5% to -11.8%) |
| February 2022 | 219.8 | 188.4 | -14.3% (-19.9% to -8.5%) |
| March 2022 | 234.0 | 202.0 | -13.6% (-19.1% to -8.0%) |
| April 2022 | 232.2 | 190.6 | -17.9% (-23.2% to -12.5%) |
| May 2022 | 230.4 | 187.4 | -18.6% (-23.9% to -13.2%) |
| June 2022 | 227.3 | 201.5 | -11.3% (-17.0% to -5.5%) |

**Supplemental Table 12: Projected and Actual Benzodiazepine Dispensing in Residents 0 to 24 years of age, Income Quintile 5, April 2020 to June 2022**

| **Month** | **Projected Rate of benzodiazepine dispensing in absence of COVID pandemic (individuals per 100,000)** | **Actual Rate of benzodiazepine dispensing during COVID pandemic (individuals per 100,000)** | **Relative percent change, actual versus projected benzodiazepine dispensing (95% confidence interval** |
| --- | --- | --- | --- |
| April 2020 | 247.8 | 192.9 | -22.2% (-27.1% to -17.1%) |
| May 2020 | 242.3 | 183.0 | -24.4% (-29.3% to -19.4%) |
| June 2020 | 239.7 | 208.3 | -13.1% (-18.5% to -7.5%) |
| July 2020 | 236.5 | 210.8 | -10.8% (-16.4% to -5.1%) |
| August 2020 | 248.0 | 213.6 | -13.9% (-19.2% to -8.4%) |
| September 2020 | 236.8 | 216.2 | -8.7% (-14.3% to -2.8%) |
| October 2020 | 246.0 | 220.0 | -10.5% (-16.0% to -4.9%) |
| November 2020 | 244.5 | 216.8 | -11.3% (-16.8% to -5.7%) |
| December 2020 | 246.8 | 215.7 | -12.5% (-17.9% to -7.0%) |
| January 2021 | 247.7 | 215.1 | -13.1% (-18.5% to -7.6%) |
| February 2022 | 237.6 | 192.3 | -19.1% (-24.2% to -13.7%) |
| March 2021 | 254.2 | 220.8 | -13.1% (-18.4% to -7.7%) |
| April 2021 | 254.3 | 209.1 | -17.7% (-22.8% to -12.5%) |
| May 2021 | 248.8 | 212.1 | -14.7% (-20.0% to -9.3%) |
| June 2021 | 246.2 | 222.6 | -9.6% (-15.1% to -3.9%) |
| July 2021 | 243.0 | 203.8 | -16.1% (-21.4% to -10.7%) |
| August 2021 | 254.6 | 209.8 | -17.6% (-22.7% to -12.4%) |
| September 2021 | 243.3 | 211.9 | -12.9% (-18.3% to -7.3%) |
| October 2021 | 252.6 | 205.4 | -18.6% (-23.7% to -13.4%) |
| November 2021 | 251.0 | 209.1 | -16.7% (-21.8% to -11.4%) |
| December 2021 | 253.3 | 199.4 | -21.3% (-26.2% to -16.2%) |
| January 2022 | 254.2 | 199.2 | -21.6% (-26.5% to -16.6%) |
| February 2022 | 244.1 | 194.3 | -20.4% (-25.5% to -15.2%) |
| March 2022 | 260.7 | 219.0 | -16.0% (-21.1% to -10.7%) |
| April 2022 | 260.8 | 206.0 | -21.0% (-25.9% to -16.0%) |
| May 2022 | 255.3 | 202.6 | -20.7% (-25.6% to -15.5%) |
| June 2022 | 252.7 | 213.9 | -15.3% (-20.6% to -9.9%) |

**Supplemental Table 13: Projected and Actual Benzodiazepine Dispensing in Children and Youth 10 to 14 years of age, April 2020 to June 2022**

| **Month** | **Projected Rate of benzodiazepine dispensing in absence of COVID pandemic (individuals per 100,000)** | **Actual Rate of benzodiazepine dispensing during COVID pandemic (individuals per 100,000)** | **Relative percent change, actual versus projected benzodiazepine dispensing (95% confidence interval** |
| --- | --- | --- | --- |
| April 2020 | 69.6 | 49.2 | -29.2% (-38.0% to -19.9%) |
| May 2020 | 69.3 | 47.5 | -31.5% (-40.1% to -22.5%) |
| June 2020 | 66.6 | 57.8 | -13.1% (-23.5% to -2.1%) |
| July 2020 | 65.2 | 58.5 | -10.4% (-21.1% to -0.96%) |
| August 2020 | 69.3 | 58.1 | -15.9% (-25.9% to -5.4%) |
| September 2020 | 75.6 | 74.0 | -2.1% (-12.7% to 9.2%) |
| October 2020 | 70.2 | 70.4 | 0.34% (-10.9% to 12.2%) |
| November 2020 | 69.1 | 64.4 | -6.8% (-17.5% to 4.5%) |
| December 2020 | 65.2 | 59.4 | -8.7% (-19.6% to 2.8%) |
| January 2021 | 71.0 | 62.8 | -11.4% (-21.65 to -0.64%) |
| February 2022 | 67.3 | 59.7 | -11.3% (-21.7% to -0.23%) |
| March 2021 | 70.2 | 69.5 | -0.86% (-12.3% to 10.6%) |
| April 2021 | 71.0 | 61.7 | -13.1% (-23.1% to -2.5%) |
| May 2021 | 70.8 | 68.9 | -2.6% (-13.5% to 9.0%) |
| June 2021 | 68.1 | 73.8 | 8.5% (-3.5% to 21.3%) |
| July 2021 | 66.7 | 71.2 | 6.8% (-5.2% to 19.6%) |
| August 2021 | 70.7 | 73.0 | 3.2% (-8.1% to 15.3%) |
| September 2021 | 77.1 | 89.0 | 15.6% (3.7% to 28.1%) |
| October 2021 | 71.7 | 76.3 | 6.5% (-5.0% to 18.8%) |
| November 2021 | 70.6 | 76.6 | 8.7% (-3.2% to 21.2%) |
| December 2021 | 66.6 | 72.0 | 8.3% (-3.8% to 21.2%) |
| January 2022 | 72.4 | 66.8 | -7.6% (-18.0% to 3.3%) |
| February 2022 | 68.8 | 66.3 | -3.5% (-14.4% to 8.1%) |
| March 2022 | 71.7 | 74.3 | 3.8% (-7.5% to 15.9%) |
| April 2022 | 72.5 | 67.5 | -6.8% (-17.2% to 4.2%) |
| May 2022 | 72.3 | 68.2 | -5.6% (-16.2% to 5.5%) |
| June 2022 | 69.6 | 71.2 | 2.4% (-9.0% to 14.5%) |

**Supplemental Table 14: Projected and Actual Benzodiazepine Dispensing in Children and Youth 15 to 19 years of age, April 2020 to June 2022**

| **Month** | **Projected Rate of benzodiazepine dispensing in absence of COVID pandemic (individuals per 100,000)** | **Actual Rate of benzodiazepine dispensing during COVID pandemic (individuals per 100,000)** | **Relative percent change, actual versus projected benzodiazepine dispensing (95% confidence interval** |
| --- | --- | --- | --- |
| April 2020 | 330.2 | 243.5 | -26.2% (-30.5% to -22.0%) |
| May 2020 | 332.7 | 241.4 | -27.4% (-31.6% to -23.2%) |
| June 2020 | 325.8 | 276.1 | -15.3% (-19.9% to -10.4%) |
| July 2020 | 313.2 | 275.2 | -12.1% (-17.0% to -7.1%) |
| August 2020 | 324.2 | 278.4 | -14.1% (-18.8% to -9.3%) |
| September 2020 | 319.0 | 284.5 | -10.8% (-15.7% to -5.8%) |
| October 2020 | 332.9 | 286.7 | -13.9% (-18.5% to -9.1%) |
| November 2020 | 326.8 | 278.5 | -14.8% (-19.5% to -10.0%) |
| December 2020 | 324.5 | 274.5 | -15.4% (-20.0% to -10.6%) |
| January 2021 | 333.5 | 283.4 | -15.0% (-19.7% to -10.3%) |
| February 2022 | 320.7 | 257.3 | -19.8% (-24.3% to -15.1%) |
| March 2021 | 338.2 | 296.1 | -12.4% (-17.1% to -7.6%) |
| April 2021 | 338.7 | 274.8 | -18.9% (-23.3% to -14.3%) |
| May 2021 | 341.1 | 287.4 | -15.7% (-20.3% to -11.1%) |
| June 2021 | 334.3 | 298.1 | -10.8% (-15.6% to -5.9%) |
| July 2021 | 321.6 | 275.3 | -14.4% (-19.1% to -9.6%) |
| August 2021 | 332.7 | 277.7 | -16.5% (-21.1% to -11.8%) |
| September 2021 | 327.5 | 287.9 | -12.1% (-16.8% to -7.2%) |
| October 2021 | 341.3 | 272.7 | -20.1% (-24.5% to -15.6%) |
| November 2021 | 335.3 | 280.4 | -16.3% (-20.9% to -11.7%) |
| December 2021 | 332.9 | 259.8 | -21.9% (-26.3% to -17.5%) |
| January 2022 | 342.0 | 254.9 | -25.5% (-29.6% to -21.2%) |
| February 2022 | 329.2 | 249.0 | -24.3% (-28.6% to -19.9%) |
| March 2022 | 346.7 | 284.4 | -17.9% (-22.3% to -13.4%) |
| April 2022 | 347.1 | 253.5 | -26.9% (-31.0% to -22.8%) |
| May 2022 | 349.6 | 251.5 | -28.0% (-32.0% to -24.0%) |
| June 2022 | 342.7 | 272.2 | -20.6% (-24.9% to -16.1%) |

**Supplemental Table 15: Projected and Actual Benzodiazepine Dispensing in Children and Youth 20 to 24 years of age, January 2021 to June 2022**

| **Month** | **Projected Rate of benzodiazepine dispensing in absence of COVID pandemic (individuals per 100,000)** | **Actual Rate of benzodiazepine dispensing during COVID pandemic (individuals per 100,000)** | **Relative percent change, actual versus projected benzodiazepine dispensing (95% confidence interval** |
| --- | --- | --- | --- |
| January 2021 | 654.6 | 604.1 | -7.7% (-11.1% to -4.3%) |
| February 2022 | 618.2 | 565.1 | -8.6% (-12.0% to -5.1%) |
| March 2021 | 662.7 | 625.5 | -5.6% (-9.0% to -2.2%) |
| April 2021 | 651.6 | 587.8 | -9.8% (-13.1% to -6.5%) |
| May 2021 | 635.4 | 585.1 | -7.9% (-11.3% to -4.5%) |
| June 2021 | 629.4 | 581.6 | -7.6% (-11.1% to -4.1%) |
| July 2021 | 632.5 | 563.7 | -10.9% (-14.2% to -7.5%) |
| August 2021 | 646.9 | 557.2 | -13.9% (-17.1% to -10.6%) |
| September 2021 | 626.7 | 560.6 | -10.5% (-13.9% to -7.1%) |
| October 2021 | 643.3 | 548.6 | -14.7% (-17.9% to -11.5%) |
| November 2021 | 635.6 | 570.5 | -10.2% (-13.6% to -6.9%) |
| December 2021 | 630.1 | 540.6 | -14.2% (-17.5% to -10.9%) |
| January 2022 | 665.8 | 554.2 | -16.7% (-19.9% to -13.6%) |
| February 2022 | 629.4 | 543.5 | -13.6% (-17.0% to -10.3%) |
| March 2022 | 673.9 | 592.3 | -12.1% (-15.3% to -8.8%) |
| April 2022 | 662.8 | 552.9 | -16.6% (-19.7% to -13.4%) |
| May 2022 | 646.6 | 548.4 | -15.2% (-18.4% to -11.9%) |
| June 2022 | 640.6 | 556.6 | -13.1% (-16.4% to -9.8%) |

**Supplemental Figure 1: Impact of COVID-19 (April 2020) on monthly rates of benzodiazepine dispensing among Ontario residents between the ages of 0 and 24, January 2013 to June 2022**


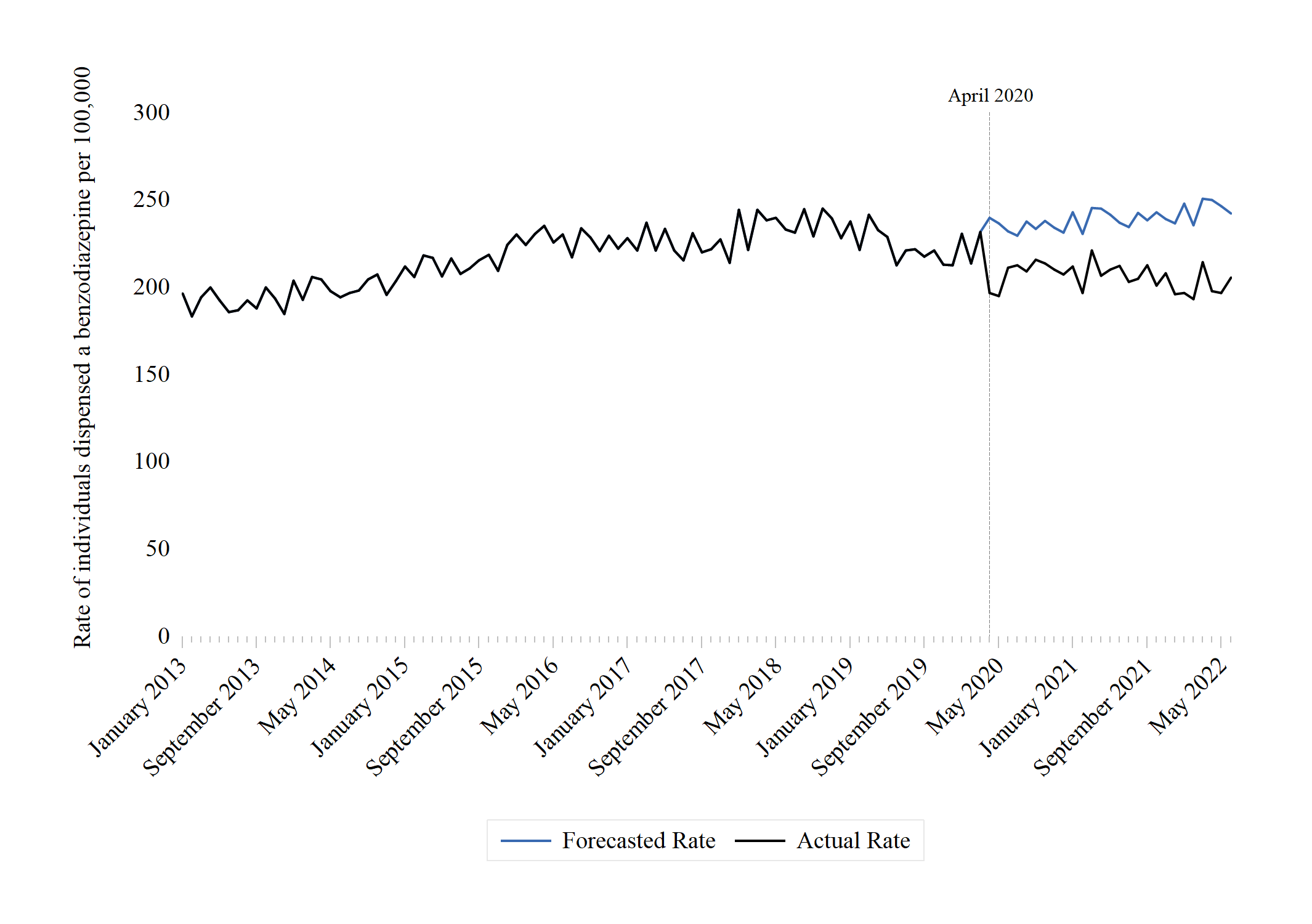


**Supplemental Figure 2: Impact of COVID-19 (April 2020) on monthly rates of benzodiazepine dispensing among Ontario residents between the ages of 10 and 14, January 2013 to June 2022**


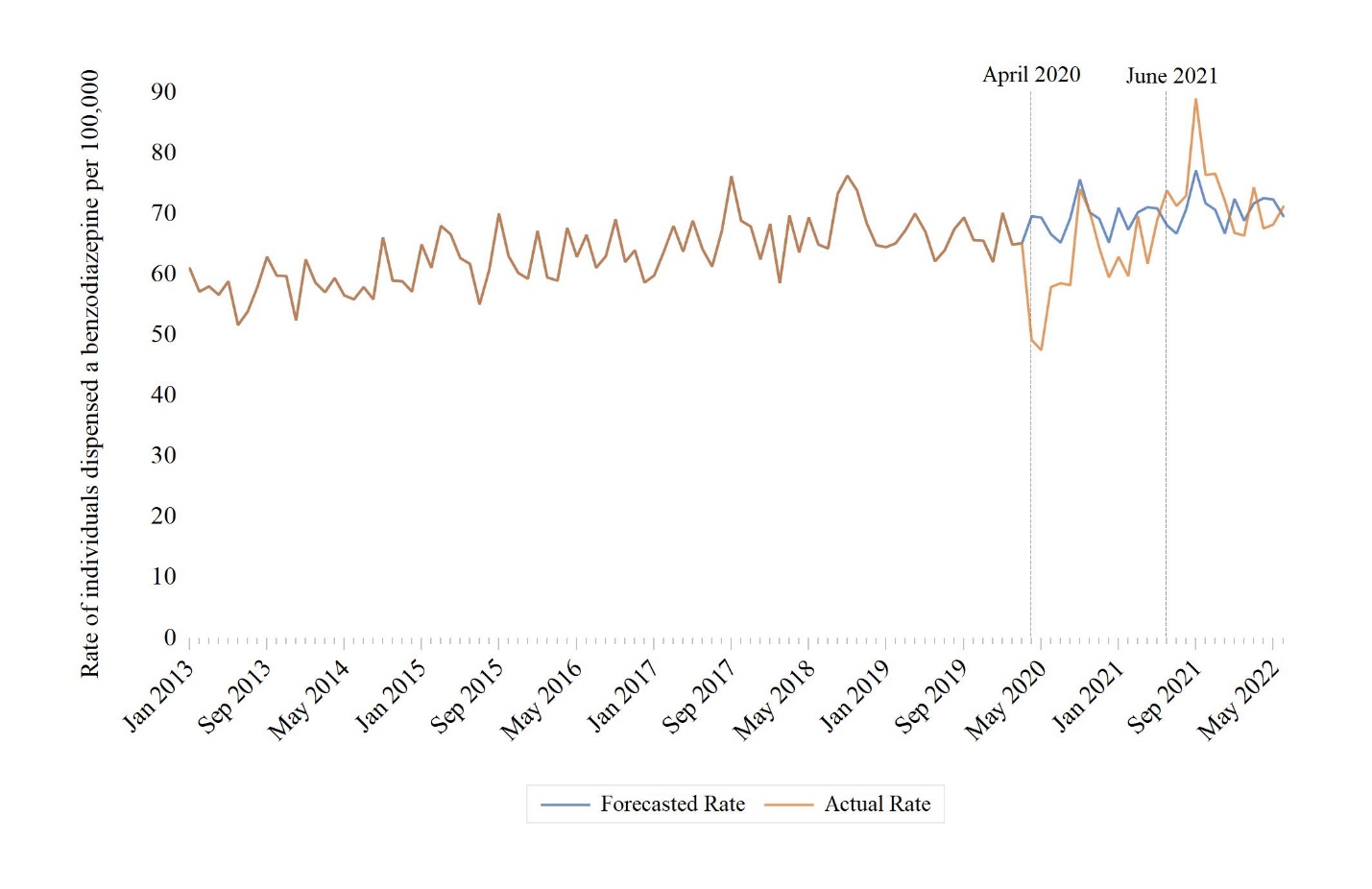

Supplement: Supplementary file 1 — Supplementary Material 1 [file 787_2024_2531_MOESM1_ESM.docx]
